# Wolframin‐1–expressing neurons in the entorhinal cortex propagate tau to neocortical brain regions under the amyloid pathology influence

**DOI:** 10.1002/alz70855_106794

**Published:** 2025-12-24

**Authors:** Seiko Ikezu, Arun Reddy Ravula, Ayaka Tatsumoto, Brendan Gibbs, Stephanie Radhakishun, Justice Ellison, Alejandro Longtree‐Preciado, Nibedita Basu Ray, Tsuneya Ikezu

**Affiliations:** ^1^ Mayo Clinic Florida, Jacksonville, FL, USA

## Abstract

**Background:**

We have recently reported that wolframin‐1‐expressing (Wfs1^+^) pyramidal neurons in the entorhinal cortex layer II (ECII) that projects to the CA1 propagate phosphorylated tau (pTau) via the temporoammonic pathway, mimicking early stages of tau pathology in AD (ECII‐CA1 tau mice). We examined if amyloid pathology and human tau expression with this mouse model may change the character and distribution of tau transfer. Furthermore, using a chemogenetic approach, we aimed to determine the effect of neuronal excitability on tau transfer to the hippocampal regions in mouse brains.

**Method:**

Cre‐inducible AAV expression P301L tau mutant (AAV‐Flex‐P301Ltau) was injected into the EC II region of APP NL‐G‐F human MAPT double knockin (APP^NL‐G‐F^: TAUKI) mice crossed with Wfs1‐Cre and Wfs1‐Cre mice at 7 months of age. Tau propagation was evaluated in the hippocampal and cortex regions by immunofluorescence using HT7 (human tau) and AT8 (pS^202^/pT^205^ tau) mAbs at one‐ and 3‐months post‐injection. AAV‐Flex‐P301Ltau was co‐injected with Cre‐inducible AAV expressing hM3D(Gq) or hM4D(Gi) DREADD in the ECII in Wfs1‐Cre mice at 4 months of age, followed by subcutaneous infusion of clozapine *N*‐oxide or saline via osmotic pumps for 28 days. Immunofluorescence against HT7 mAb was performed at one‐month post‐injection.

**Result:**

We observed robust HT7 positivity in the CA1 and subiculum in Wfs1‐Cre mice after AAV‐Flex‐P301Ltau injection, although there was little AT8 staining at both one‐ and 3‐month. Interestingly, APP^NL‐G‐F^:TauKI:Wfs1‐Cre mice showed strong HT7 and AT8 positivity in the subiculum, but not in CA1 at 1‐month. Notably, *p*‐tau further advanced into the visual cortex (VC) region at 3‐month in APP^NL‐G‐F^:TauKI:Wfs1‐Cre mice, while neither HT7 nor AT8 was positive in the VC in Wfs1‐Cre mice. Increased or suppressed neuronal excitability in the medial ECII pyramidal neurons by DREADD approach significantly induced or reduced tau propagation to the CA1 compared to the control group at one‐month post‐injection, respectively.

**Conclusion:**

Our study demonstrates that APP^NL‐G‐F^:TAUKI:Wfs1‐Cre mice injected with P301L tau in the ECII recapitulates advanced‐stage AD pathology with amyloid beta navigating tau to the neocortical region and enhancing the maturity of tau pathology. Furthermore, ECII neuronal excitability regulates tau transfer efficiency from the ECII to CA1 region.

